# Replication of 10 novel loci involved in human plasma protein N-glycosylation using MALDI-MS and UHPLC-FD data

**DOI:** 10.1093/glycob/cwag029

**Published:** 2026-04-20

**Authors:** Anna Timoshchuk, Annemieke Naber, Roderick Slieker, Anna Soplenkova, Denis E Maslov, Nadezhda A Potapova, Simone Nicolardi, P J M Elders, Eric J G Sijbrands, Sodbo Sharapov, Leen M 't Hart, Mandy van Hoek, Manfred Wuhrer, Yurii S Aulchenko

**Affiliations:** MSU Institute for Artificial Intelligence, Lomonosov Moscow State University, Lomonosovsky Prospekt, 37, Building 1, 119192, Moscow, Russia; Department of Internal Medicine, Erasmus Medical Center, University Medical Center Rotterdam, Dr. Molewaterplein 40, 3015 GD, Rotterdam, the Netherlands; Department of Epidemiology and Data Science, Amsterdam Public Health Institute, Amsterdam Cardiovascular Sciences, De Boelelaan 1117, 1081 HV, Amsterdam, the Netherlands; Department of Cell and Chemical Biology, Leiden University Medical Center, Einthovenweg 20, 2333 ZC, Leiden, the Netherlands; MSU Institute for Artificial Intelligence, Lomonosov Moscow State University, Lomonosovsky Prospekt, 37, Building 1, 119192, Moscow, Russia; MSU Institute for Artificial Intelligence, Lomonosov Moscow State University, Lomonosovsky Prospekt, 37, Building 1, 119192, Moscow, Russia; Institute of Cytology and Genetics, Siberian Branch of Russian Academy of Sciences, rospekt Akademika Lavrent’yeva, 10, 630090, Novosibirsk, Russia; Center for Proteomics and Metabolomics, Leiden University Medical Center, Albinusdreef 2, 2333 ZA, Leiden, the Netherlands; Department of General Practice, Amsterdam UMC, Location Vrije Universiteit, Meibergdreef 15, 1105AZ, Amsterdam, the Netherlands; Amsterdam Public Health Research Institute, Van der Boechorststraat 7, 1081 BT, Amsterdam, the Netherlands; Department of Internal Medicine, Erasmus Medical Center, University Medical Center Rotterdam, Dr. Molewaterplein 40, 3015 GD, Rotterdam, the Netherlands; MSU Institute for Artificial Intelligence, Lomonosov Moscow State University, Lomonosovsky Prospekt, 37, Building 1, 119192, Moscow, Russia; Department of Epidemiology and Data Science, Amsterdam Public Health Institute, Amsterdam Cardiovascular Sciences, De Boelelaan 1117, 1081 HV, Amsterdam, the Netherlands; Department of Cell and Chemical Biology, Leiden University Medical Center, Einthovenweg 20, 2333 ZC, Leiden, the Netherlands; Department of Internal Medicine, Erasmus Medical Center, University Medical Center Rotterdam, Dr. Molewaterplein 40, 3015 GD, Rotterdam, the Netherlands; Center for Proteomics and Metabolomics, Leiden University Medical Center, Albinusdreef 2, 2333 ZA, Leiden, the Netherlands; MSU Institute for Artificial Intelligence, Lomonosov Moscow State University, Lomonosovsky Prospekt, 37, Building 1, 119192, Moscow, Russia; Institute of Cytology and Genetics, Siberian Branch of Russian Academy of Sciences, rospekt Akademika Lavrent’yeva, 10, 630090, Novosibirsk, Russia

**Keywords:** genetic control of N-glycosylation, GWAS, MALDI-MS, N-glycans, UHPLC-FD

## Abstract

N-glycans are essential components of glycoproteins, influencing their properties and functions. While biochemical pathways of glycosylation are well-characterized, their genetic regulation remains poorly understood. This study utilizes matrix-assisted laser desorption/ionization-mass spectrometry (MALDI-MS) and ultra-high performance liquid chromatography-fluorescence detection (UHPLC-FD) to strengthen replication and further characterize previously identified genome-wide association signals for the total human plasma N-glycome (TPNG). Univariate and multivariate genetic association meta-analyses involved 3385 samples across 143 N-glycome traits from the Hoorn Diabetes Care System and DiaGene cohorts as well as 3224 samples across 117 N-glycome traits from TwinsUK, CEDAR, QMDiab and SABRE cohorts. We successfully replicated ten previously identified but not replicated glycosylation quantitative trait loci (glyQTLs) and prioritized five high-confidence putative causal genes, including the glycosyltransferase *MGAT4B* and inflammation-related genes – *C3* and *FCGR2B.* The linkage-specific sialic acid derivatization in MALDI-MS enabled delineation of genetic effects on α2,3- and α2,6-sialylation. Mass spectrometry analysis, triggered and guided by association to a locus containing *B3GAT1* glucuronosyltransferase, provided evidence for hexuronic acid-containing glycans in human blood plasma. These findings advance our understanding of the genetic regulation of protein N-glycosylation and highlight the complementarity of different analytical approaches in glycomics research.

## Introduction

N-glycans are irregular polymers attached to polypeptide structures, forming glycoproteins with a glycosidic bond to the asparagine (Asn) side chain (Varki et al. 2022). Glycosylation impacts the physical and chemical characteristics of proteins, along with their biological roles. Changes in protein glycosylation are associated with a number of human diseases, and glycans are seen as biomarkers and essential components of treatments (Reily et al. 2019; Lauc and Trbojević-Akmačić 2021). Although the biochemical pathways of glycosylation are well understood (Varki et al. 2022), genetic and regulatory networks controlling glycan diversity, tissue-specific expression, and disease-related variation remain poorly understood. The lack of understanding of mechanisms governing the variation in the glycome and connecting it to human health and disease delays the progress in the clinical application of human glycobiology (Reily et al. 2019; Lauc and Trbojević-Akmačić 2021).

Quantitative genetics provides a powerful tool to study genetic regulation of protein N-glycosylation. Genome-wide association studies (GWASs) are a widely used approach to map genetic loci involved in the control of multifactorial diseases and complex traits. In an N-glycosylation GWAS, variation in observed phenotypes – levels of different N-glycan species – is statistically related to variation in genetic markers across the genome. A typical GWAS identifies a set of loci associated with the phenotype. These loci are considered as candidate regions for further experimental follow-up and functional validation (Abdellaoui et al. 2023). In the most recent genome-wide association study (GWAS) of total plasma N-glycome (TPNG), we used an ultra-high performance liquid chromatography-fluorescence detection (UHPLC-FD) to measure N-glycans in about 10,000 analyzed samples (Sharapov et al. 2025). This study discovered 59 TPNG glycan quantitative trait loci (glyQTLs), 40 of which were replicated in an independent cohort of 3224 individuals.

Understanding the spectrum of glycans associated with a specific locus is crucial for interpreting the association of the locus, including prioritization of candidate genes and formulating hypotheses about mechanisms through which genetic variation in the locus affects the phenotype. For instance, when a locus contains a glycosyltransferase gene, and the variation associated with this locus concerns N-glycans that are expected to be affected by the glycosyltransferase, this strongly supports the glycosyltransferase as the primary candidate gene in that region. Examples include association of the *FUT8*-containing region with core-fucosylated N-glycans and their substrates, and the *MGAT3* region with bisecting GlcNAc (Timoshchuk et al. 2023). Phenotypic variation can indicate the tissue of action of the gene, e.g. a locus affecting tri- and tetra-antennary structures of TPNG is probably acting in the liver, as these structures are predominantly expressed on liver-secreted glycoproteins (Timoshchuk et al. 2023).

Conversely, a mismatch between expected action of a candidate gene and a strong biological prior (such as a glycosyltransferase) and the phenotypic effects of the locus may suggest an alternative candidate gene, a complex, e.g. indirect, mechanism of action, or a methodological artifact. For instance, the *B3GAT1* gene, which encodes a key enzyme involved in the glucuronyl transfer reaction, is a candidate for being a causal gene in one of the first loci linked to TPNG (Huffman et al. 2011). However, glucuronic acid monomers are not typically found in the N-glycome of human blood plasma. These associations triggered mass spectrometric analysis of the associated TPNG peaks which confirmed the presence of hexuronic acid-containing N-glycans (Huffman et al. 2011). Another example concerns, the *ABO* locus that encodes glycosyltransferase enzymes known to convert H antigen substrates into blood group antigens. However, plasma glycoproteins lack the H antigen substrate, making it unlikely that the genetic association between the *ABO* locus and TPNG is driven directly by *ABO* glycosyltransferase activity, suggesting instead an indirect or alternative mechanistic explanation.

Utilizing different analytical techniques allows for a more detailed understanding of the glycan spectrum associated with specific loci, supporting the goal of deciphering the biology of associated loci. Most previous studies of quantitative genetics of glycans employed HPLC (Timoshchuk et al. 2023), while other high-throughput technologies for TPNG exist (Reiding et al. 2019). These include multiplexed capillary gel electrophoresis with laser-induced fluorescence detection (xCGE-LIF) (Maslov et al. 2024), and matrix-assisted laser desorption ionization mass spectrometry (MALDI-MS). UHPLC-FD and xCGE-LIF technologies allow separating structural isomers of N-glycans, providing branch-specific information, that is, a separation between the 3-arm and 6-arm isomers of glycan species (for example, FA2[3]G1 and FA2[6]G1). MALDI-MS is unable to differentiate the structural isomers.

In the current study, we took advantage of analyzing data generated by UHPLC-FD and MALDI-MS. One of the unique features of the latter method is linkage-specific separation of α2,3- and α2,6-sialylation due to specific sample preprocessing. This distinction is particularly valuable since previous studies demonstrated that, these two sialylation types may have different, and even opposite associations with diseases. For example, diabetes and liver fibrosis in metabolic dysfunction-associated steatotic liver disease (MASLD) are associated with higher α2,6-sialylation and lower α2,3-sialylation (Blood N-glycomic signature of fibrosis in MASLD shows low levels of global α2,3-sialylation | medRxiv 2026; Dotz et al. 2018).

In the present study, our objectives were to replicate the association of the 19 loci, previously reported to be associated with TPNG at GWAS significance level, but not yet replicated (Sharapov et al. 2021; Sharapov et al. 2025), and to prioritize genes, likely playing a role in regulation of N-glycosylation of plasma proteins, in newly replicated loci. Further, we aimed to utilize UHPLC-FD and MALDI-MS N-glycan spectra to provide extended annotation of the phenotypic effects, i.e. associated N-glycans, of the loci with confirmed association with TPNG (i.e. replicated here and elsewhere) (Sharapov et al. 2021; Sharapov et al. 2025).

## Results

### Replication

Two groups of cohorts were used for replication. The first one involved 3224 samples from several cohorts phenotyped with UHPLC-FD across 117 N-glycome traits, that were used for replication in Sharapov et al. (Sharapov et al. 2025). The second group is unique to this study and includes 3385 samples from the Hoorn Diabetes Care System and DiaGene cohorts, phenotyped for 143 N-glycome traits with MALDI-MS (Supplementary Table 1a-c). A per-trait, per-locus meta-analysis was applied separately to cohorts within the two groups. As we cannot fully match the N-glycan traits measured by MALDI-MS (Supplementary Table 2a and b) and UHPLC-FD (Supplementary Table 2c and d), for each of the 19 glyQTLs not replicated in Sharapov et al., we applied the Cauchy aggregation test (Liu and Xie 2020) to combine P-values across all glycan traits, resulting in a single p-value per locus per group. We then conducted a Fisher product meta-analysis (Brown 1975) for Cauchy aggregated p-values from UHPLC-FD and MALDI-MS groups, resulting in 19 replication p-values (Supplementary Table 3). A locus was considered replicated if the Fisher product meta-analysis p-value exceeded the threshold of 0.05/19 = 2.63 × 10^−3^, where 19 is the number of glyQTLs analyzed here.

We replicated 10 loci (containing *FCGR2B, NDUFB4P4, PCCB, RNF168, MGAT4B, GRID1P, ODF1, OVOL1, RPLP2P4, C3* genes) out of 19 tested for replication (Table 1). The details of the replication procedure are presented in the Supplementary Table 3.

**Table 1:** Results of the replication of 19 loci not replicated in the study by Sharapov et al. (Sharapov et al. 2025).

**Index SNP**	**Chr**	**Pos**	**EA**	**RA**	**Gene**	**P Cauchy UHPLC-FD**	**P Cauchy MALDI-MS**	**P Fisher**
**rs34881159**	**1**	**161,590,503**	**A**	**T**	***FCGR2B***	**4,25E-02**	**4,59E-08**	**4,11E-08**
**rs10188206**	**2**	**26,142,632**	**A**	**T**	***NDUFB4P4***	**6,79E-03**	**1,62E-02**	**1,11E-03**
**rs523118**	**3**	**135,965,888**	**G**	**T**	***PCCB***	**1,84E-06**	**3,11E-04**	**1,27E-08**
**rs7647487**	**3**	**196,206,175**	**C**	**T**	***RNF168***	**1,42E-03**	**4,80E-02**	**7,21E-04**
**rs76360119**	**5**	**179,183,793**	**A**	**G**	***MGAT4B***	**2,84E-05**	**2,34E-05**	**1,47E-08**
rs9352006	6	74,238,815	A	G	*MBD4*	1,19E-01	1,77E-01	1,03E-01
**rs2881699**	**7**	**6,544,886**	**C**	**G**	***GRID2IP***	**1,67E-05**	**2,15E-02**	**5,68E-06**
**rs2511737**	**8**	**103,580,527**	**A**	**C**	***ODF1***	**4,76E-02**	**1,73E-04**	**1,05E-04**
rs7866188	9	94,681,588	A	C	*ROR2*	3,89E-01	1,03E-01	1,69E-01
rs2497318	10	94,432,000	C	T	*EIF2S2P3*	1,13E-01	8,07E-03	7,32E-03
**rs10896045**	**11**	**65,555,524**	**A**	**G**	***OVOL1***	**1,87E-02**	**3,80E-03**	**7,48E-04**
rs667633	11	114,366,732	C	T	*NXPE2*	4,10E-01	2,30E-01	3,17E-01
**rs1150975**	**12**	**32,052,422**	**A**	**C**	***RPLP2P4***	**1,68E-02**	**1,18E-03**	**2,34E-04**
rs7402780	15	44,336,044	A	G	*FRMD5*	6,15E-01	1,79E-02	6,07E-02
rs11071549	15	60,941,324	G	T	*RORA*	9,12E-01	8,91E-01	9,81E-01
rs2305479	17	38,062,217	C	T	*GSDMB*	7,22E-03	8,46E-02	5,13E-03
rs56214516	17	43,836,953	A	C	*CRHR1*	2,38E-01	1,09E-02	1,81E-02
**rs11085197**	**19**	**6,713,175**	**C**	**G**	***C3***	**5,54E-02**	**2,64E-25**	**8,85E-25**
rs7412	19	45,412,079	C	T	*APOE4*	5,73E-01	2,10E-03	9,30E-03

Ten loci (in bold) are replicated in the current study with a threshold of the Fisher product meta-analysis p-value of 2.63E-03. Full results are presented in Supplementary Table 3. Chr—chromosome; Pos—position on chromosome according to GRCh37.p13 assembly; EA—Effect allele; RA—Reference allele; Gene—Nearest gene or gene prioritized in this work or in previous GWAS for human plasma protein N-glycome; P Cauchy UHPLC-FD—Cauchy aggregated P-value across all UHPLC-FD-measured glycan traits; P Cauchy MALDI-MS—Cauchy aggregated P-value across all MALDI-MS-measured glycan traits; P Fisher–Fisher product meta-analysis P-value.

### Prioritization of causal genes for protein N-glycosylation

We used several approaches to prioritize the most likely effector genes for the ten loci replicated in this work: prioritization of genes encoding glycosyltransferases; genes causing congenital disorders of glycosylation (CDG); colocalization of glyQTLs with gene expression QTLs (eQTLs) in liver and whole blood, and loci associated with blood plasma protein levels (pQTLs); annotation of putative causal variants affecting protein structure; enrichment of gene sets and tissue-specific expression; and prioritization of the nearest gene (see Methods).

**Figure 1:**
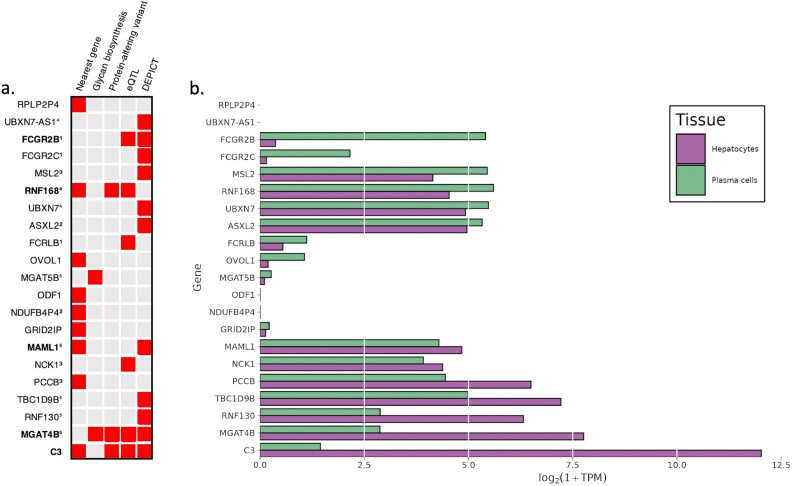
Candidate genes. a) Predictors indicating 22 candidate genes. The identical superscripts denote candidate genes inside one locus. Five high-confidence putative causal genes are highlighted in bold. Full details of the gene prioritization are presented in Supplementary Table 4a. b) Gene expression of the candidate genes in two relevant cell types: Hepatocytes and plasma cells. Expression levels are represented as the median logarithm of transcripts per million. The median expression of the *ODF1* and *NDUFB4P4* genes is exactly zero for both tissues (plasma and hepatocytes). However, for *ODF1*, the plasma contains samples with non-zero expression (maximum logTPM = 0.395). The data for hepatocytes (*n* = 513) and plasma cells (*n* = 53) samples were obtained from the ARCHS4 portal (Lachmann et al. 2018).

In total, 22 candidate genes showed at least one indication for prioritization in 10 replicated glyQTLs (Fig. 1a, Supplementary Table 4a). Among these, we identified two genes in the same locus encoding glycosyltransferases (*MGAT5B, MGAT4B*). In 3 genes (*RNF168*, *MGAT4B*, *C3*), associated variants were either coding or were in strong LD with the variants coding for potentially deleterious amino acid changes (annotated by Variant Effect Predictor, VEP) (McLaren et al. 2016) (Supplementary Table 4b-d). The DEPICT gene prioritization tool (Pers et al. 2015) provided evidence of prioritization for 12 genes in 6 loci at FDR < 0.2 (Supplementary Table 4e). The Summary data-based Mendelian Randomization analysis followed by the Heterogeneity in Dependent Instruments (SMR/HEIDI) approach (Zhu et al. 2016) indicated that total plasma N-glycosylation–associated variants in two loci possibly had pleiotropic effects on transcription of three genes (*FCGR2B*, *FCGRLB, NCK1*) in relevant tissues (Supplementary Table 4f). We didn’t prioritize any genes based on literature annotations related to CDG genes, and none of genes showed colocalization with whole blood pQTLs (Supplementary Table 4g).

We required a candidate gene to be expressed in liver or plasma cells, because these two tissues are the main producents of N-glycosylation (Clerc et al. 2016) (Fig. 1b). We prioritized it as potentially causal if at least two pieces of evidence supported it. This resulted in prioritizing five high-confidence putative causal genes: *FCGR2B, RNF168, MAML1, MGAT4B,* and *C3*), highlighted in bold in Fig. 1a, which will be the focus of our subsequent discussion.

### Characterization of glyQTLs by spectrum of associated MALDI-MS N-glycan traits

We compared annotations, specifically R^2^ of association between a locus and a top trait, for all loci (Supplementary Tables 5a and b). We observed a consistency between associated spectra of glycans measured with UHPLC-FD and MALDI-MS. Below, we annotated the glycosyltransferase loci associated with MALDI-MS-derived glycan traits at genome-wide significance level *P* < 5 × 10^−8^.

The *FUT8* locus (fucosyltransferase 8, an enzyme responsible for the addition of core fucose to glycans) was significantly associated with 14 glycan traits, all of which were fucosylated structures. These included core-fucosylated di-antennary glycans (e.g. A2FGE, A2FGS, H5N4F1E2), fucosylated tri-antennary traits (A3Fa, A3L0F), and fucosylated sialylated species such as H6N5F2E2L1. Of the 44 glycan traits significantly associated with *FUT6* locus (fucosyltransferase 6, an enzyme responsible for the addition of antennary fucose to glycans), 28 were fucosylated and 16 were non-fucosylated. The strongest associations were observed for a spectrum of tri- and tetra-antennary fucosylated sialylated glycans (e.g. H7N6F1E1L3, H7N6F2E2L2, A4FGE). The *MGAT3* locus (beta-1,4-mannosyl-glycoprotein 4-beta-N-acetylglucosaminyltransferase, an enzyme responsible for adding bisecting GlcNAc to N-glycans) was significantly associated with six glycan traits. These included direct glycan compositions (H3N5F1, H4N5F1) and derived traits specifically representing bisected structures (A2FB, A2FS0B, A2FSB, A2S0B), consistent with the enzyme’s known activity. The locus containing *MGAT5* gene which encodes the enzyme (β1,6-N-acetylglucosaminyltransferase V) that catalyzes the addition of beta-1,6-N-acetylglucosamine to the alpha6-linked mannose of di-antennary glycans was significantly associated with four glycan traits. The associated traits included tri- and tetra-antennary sialylated structures (H6N5E1, H7N6E2L1) and derived traits reflecting tri-antennary glycans without fucose (A3F0S, A3S), consistent with the enzyme’s established role in generating branched, complex N-glycans. The *B4GALT1* gene encodes β1,4-galactosyltransferase, an enzyme responsible for adding galactose to N-glycans during biosynthesis. This locus was significantly associated with two glycan traits, both reflecting galactosylation of di-antennary glycans: A2FS0G (galactosylation of fucosylated non-sialylated di-antennary glycans) and A2S0G (galactosylation of non-fucosylated non-sialylated di-antennary glycans). These associations are consistent with the established role of *B4GALT1* in galactose addition. Annotation of other loci can be found in Supplementary Table 5a and b.

By derivatizing sialic acid with linkage-specific ethylation and lactonization, we could distinguish between α2,6-linked and α2,3-linked sialic acid residues based on their mass. In order to identify the benefits of sialic acid derivatizing provided by MALDI-MS compared to UHPLC-FD we used the following three benchmarking loci: *ST6GAL1*, *ST3GAL6*, *ST3GAL4* (Table 2).

**Table 2:** Annotation of the sialyltransferase loci.

Glycan Trait (MALDI-MS)	Corresponding glycan trait (UHPLC-FD)	Measurement technology	*R* ^2^ × 100 of association with *ST3GAL6*	*R* ^2^ × 100 of association with *ST3GAL4*	*R* ^2^ × 100 of association with *ST6GAL1*
sialylation per antenna within all complex glycans	the percentage of sialylated structures in total plasma glycans	UHPLC-FD	0,00	0,55	0,02
		MALDI-MS	0,00	0,11	0,03
α2,6-sialylation per antenna within diantennary glycans	the percentage of disialylated structures in total plasma glycans	UHPLC-FD	0,00	0,49	0,01
		MALDI-MS	0,05	0,18	0,00
α2,6-sialylation per antenna within tri-antennary glycans	the percentage of trisialylated structures in total plasma glycans	UHPLC-FD	0,01	0,04	0,16
		MALDI-MS	0,45	7,82	0,15
α2,6-sialylation per antenna within tetra-antennary glycans	the percentage of tetrasialylated structures in total plasma glycans	UHPLC-FD	0,10	1,05	0,02
		MALDI-MS	0,61	8,76	0,18
α2,3-sialylation per antenna within diantennary glycans	the percentage of disialylated structures in total plasma glycans	UHPLC-FD	0,00	0,49	0,01
		MALDI-MS	0,23	6,80	0,01
α2,3-sialylation per antenna within tri-antennary glycans	the percentage of trisialylated structures in total plasma glycans	UHPLC-FD	0,01	0,04	0,16
		MALDI-MS	0,64	13,99	0,07
α2,3-sialylation per antenna within tetra-antennary glycans	the percentage of tetrasialylated structures in total plasma glycans	UHPLC-FD	0,10	1,05	0,02
		MALDI-MS	0,92	12,17	0,11

Full annotation results are presented in Supplementary Table 5a and b. For R^2^ calculation, we used SNPs that showed the most significant association with N-glycome trait from (Sharapov et al. 2025).

The *ST3GAL4* locus (beta-galactoside alpha-2,3-sialyltransferase 4) was significantly associated with 39 glycan traits, primarily representing α2,3-sialylated structures. These included di-, tri-, and tetra-antennary glycans with α2,3-linked sialic acids (e.g. A2L, A3L, A4L, H5N4F1E1L1, H7N6E1L3) as well as fucosylated variants (A3FL, A4FGL). The strongest association was observed with the derived trait A3L (α2,3-sialylation per antenna within tri-antennary glycans, R^2^ = 0.140), consistent with the enzyme’s canonical activity. The *ST3GAL6* locus (beta-galactoside alpha-2,3-sialyltransferase 6) was significantly associated with a single glycan trait: A4L, which represents α2,3-sialylation per antenna within tetra-antennary glycans (R^2^ = 0.009). This association aligns with the enzyme’s role in adding α2,3-linked sialic acids to branched N-glycan structures. The *ST6GAL1* locus (beta-galactoside alpha-2,6-sialyltransferase 1) was significantly associated with four glycan traits, all reflecting α2,6-sialylation of galactosylated di-antennary glycans. These included the directly measured glycan H4N4F1E1 (a di-antennary core-fucosylated α2,6-sialylated structure, R^2^ = 0.019), its mono-sialylated variant H4N5F1E1, and the derived traits A2FSG and A2SG, which combine sialylated and non-sialylated galactosylated di-antennary glycans. These associations are consistent with the established specificity of *ST6GAL1* for di-antennary substrates.

Interestingly, on average the strength of genetic association between loci containing sialyltransferases and MALDI-MS-measured N-glycome traits was 5.4 times higher, when compared with UHPLC-FD-measured N-glycome traits (Table 2). Multiple factors or their combination can explain this difference. Firstly, the noise-attributed variance component of MALDI-MS measured traits can be lower than that in UHPLC-FD. Second, the current set of derived traits differs between MALDI-MS and UHPLC-FD, and MALDI-MS-derived traits may better reflect the enzymatic activity of sialyltransferases.

**Figure 2:**
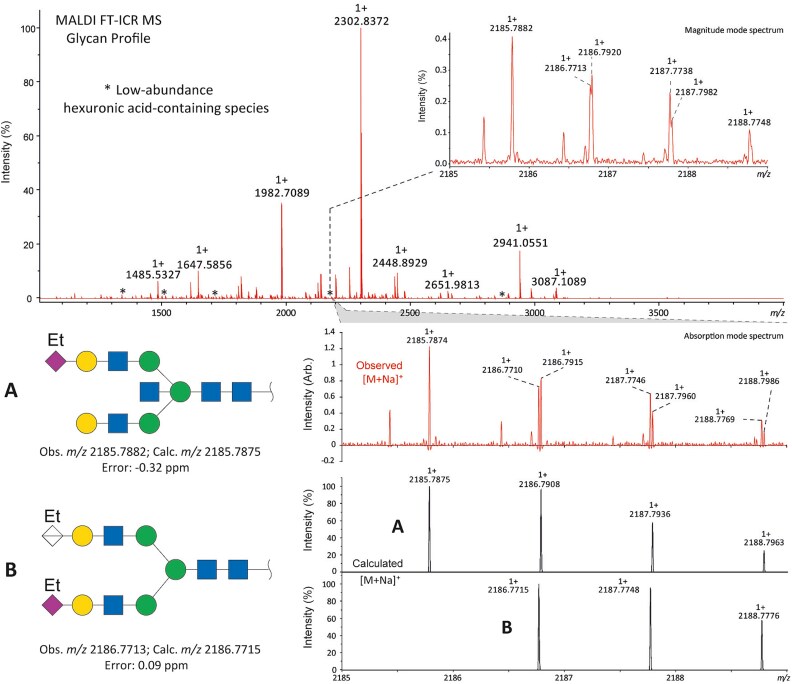
Representative ultrahigh resolution (magnitude mode) MALDI-FT-ICR-MS N-glycan profile with low-abundance hexuronic acid-containing species (top panel). Here, partially overlapping glycan species were detected (inset). Fully resolved glycan species are shown in the region between *m/*z 2185 and *m/*z 2189 of the same spectrum visualized in absorption mode. Here, an ethyl-esterified 2,6-sialylated H5N5E1 N-glycan was detected at *m/z* 2185.7874 while a ethyl-esterified hexuronic acid-containing species (i.e. H5N4E1G1) was detected at *m/z* 2186.7710. The lower two panels show the calculated spectra of species A and B. Glycan structures are depicted using standard Symbol Nomenclature for Glycans. ``Et'' indicated ethyl esterification.

The locus containing the *B3GAT1* gene encoding beta-1,3-glucuronyltransferase showed the most significant association with the glycan peak assigned as H5N5E1 (R^2^ = 0.043). In previous studies, glucuronic acid was not detected in the TPNG profile, but only in glycan profiles obtained after TPNG desialylation (Huffman et al. 2011). This unexpected association made us wonder whether the glycan peak assigned as H5N5E1 may be heterogeneous and contain an overlapping, isobaric, glucuronic acid-containing glycan signal. We therefore analyze TPNG profiles at ultrahigh resolution by absorption mode MALDI-FTICR-MS. This analysis at increased resolution confirmed the presence of the 2,6-sialylated H5N5E1 glycan (Fig. 2) at *m/z* 2185.7874 with high accuracy. A second species was detected at *m/z* 2186.7710 matched with a composition of H5N4E1G1, where G1 stands for a hexuronic acid moiety that underwent ethyl esterification during the sialic acid derivatization step (Fig. 2). The H5N4E1G1 glycan assignment was confirmed by LC–MS/MS of AB-labeled TPNG glycans, with the fragment ion at *m/z* 570.2037 indicating the presence of an antennae harboring an ethyl-esterified hexuronic acid (Supplementary Figs S1 and S2). Supplementary Fig. S2 shows that the H5N4E1G1-containing region of four additional total plasma N-glycome spectra of four healthy controls. All five TPNG samples analyzed at high resolution provided evidence for the existence of the H5N4E1G1 glycan species, warranting further investigation.

## Discussion

Our analysis of the plasma N-glycome using MALDI-MS with linkage-specific sialic acid derivatization offers novel genetic insights into the linkage-specific activity of sialyltransferases in vivo in human, with *ST3GAL4* and *ST3GAL6* associating with α2,3-sialylated glycans and *ST6GAL1* with α2,6-sialylated structures. The spectrum of glycans associated with other glycosyltransferase loci, including *FUT8*, *FUT6*, *MGAT3*, *MGAT5*, and *B4GALT1*, aligned with their known enzymatic activities. Furthermore, mass-spectra associated with variation in the *B3GAT1* locus pointed to the presence of glucuronic acid-containing glycans in the blood plasma proteome.

By applying a Cauchy aggregation test to combine data from UHPLC-FD and MALDI-MS GWAS, we successfully replicated ten previously reported loci and expanded the list of loci associated with plasma N-glycosylation by over 20% (from 40 to 50). Cauchy aggregation allows for a meta-analysis of all traits, in contrast to a meta-analysis that only examines fully matched traits. We replicated ten novel loci containing *FCGR2B, NDUFB4P4, PCCB, RNF168, MGAT4B, GRID1P, ODF1, OVOL1, RPLP2P4, C3*, and prioritized five high-confidence putative causal genes: *FCGR2B, RNF168, MAML1, MGAT4B, C3* (Table 3).

**Table 3:** Annotation of four loci containing high-confidence putative causal genes.

***FCGR2B***	***RNF168***	***MGAT4B/MAML1***	***C3***
Expressed mainly in plasma cells (Fig. 1b)	Expressed both in plasma cells and hepatocytes (Fig. 1b)	Expressed both in plasma cells and hepatocytes (Fig. 1b)	Expressed mainly in hepatocytes (Fig. 1b)
H4N4F1 (direct trait) 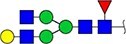 (*P* = 5,63E-10) Mostly derived from IgG (Clerc et al. 2016)	H7N6F1E1L2 (direct trait) 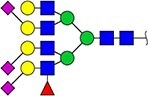 (*P* = 7,82E-04) Mostly derived from Alpha-1-acid glycoprotein (Clerc et al. 2016)	CA3 (derived trait) *Relative abundance of triantennary glycans within complex type glycans* 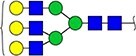 (*P* = 2,35E-05)	H9N2 (direct trait) 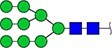 (P = 5,58E-06) Mostly derived from Apolipoprotein B-100, Immunoglobulin D (Clerc et al. 2016)
TA2FS0 (derived trait) *Fucosylated, non-sialylated diantennary species within total glycans* 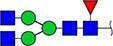 (*P* = 1,26E-09) Mostly derived from IgG (Clerc et al. 2016)	H6N5F1E1L2 (direct trait) 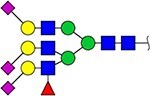 (P = 1,89E-03) Mostly derived from Alpha-1-acid glycoprotein, Alpha-1-antitrypsin, Haptoglobin, Vitronectin, Ceruloplasmin (Clerc et al. 2016)	H6N5E3 (direct trait) 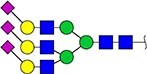 (P = 1,26E-04) Mostly derived from Apolipoprotein D, Alpha-1-acid glycoprotein, Beta-2-glycoprotein 1 (Clerc et al. 2016)	MM (derived trait) *Average number of mannoses on oligomannose type glycans* 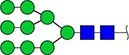 (*P* = 4,20E-04) Mostly derived from Alpha-2-macrogobulin, Immunoglobulin M, Immunoglobulin D (Clerc et al. 2016)
H4N5F1 (direct trait) 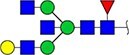 (P = 4,28E-09) Mostly derived from IgG (Clerc et al. 2016)	CA2 (derived trait) *Relative abundance of diantennary glycans within complex type glycans* 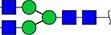 (*P* = 3,49E-03)	CA2 (derived trait) *Relative abundance of diantennary glycans within complex type glycans* 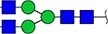 (*P* = 1,63E-04)	A2FGS (derived trait) *Sialylation per galactose within fucosylated diantennary glycans* 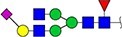 (P = 1,34E-02)

For each locus, the top three associated N-glycome traits are represented. Full annotation results are presented in Supplementary Table 5b.

The genes prioritized in these loci with high confidence add to our understanding of the major pathways involved in the regulation of plasma protein N-glycosylation, i.e. glycan biochemistry (*MGAT4B*), liver (*C3*), and B cell (*FCGR2B*) biology. The top three associations for *MGAT4B* are complex high-branched N-glycans (CA3, H6N5E3, CA2) that corresponds to the enzymatic activity of this glycosyltransferase (Varki et al. 2022) (Table 3). The *C3* locus is associated with oligomannose glycans typical of hepatocyte-produced proteins (Table 3) (Clerc et al. 2016). The *FCGR2B* locus, bearing genes for IgG receptors expressed in B-cells and other leukocytes, is associated with N-glycome traits predominantly derived from IgG (e.g. H4N4F1) (Table 3) (Clerc et al. 2016).

Indeed, *FCGR2B* encodes FcγRIIb, an inhibitory IgG receptor on B cells, basophils and monocytes that reduces phagocytosis and pro-inflammatory cytokine release by competing with activating Fcγ receptors for immune complexes. Crosslinking FcγRIIb with the B-cell receptor raises the B-cell activation threshold, lowering antibody production and affecting the plasma N-glycome composition (Smith and Clatworthy 2010). Nearby gene *FCGR2C* may also affect glycosylation, it also encodes an activating FcγR on NK cells, but unlike FcγRIIb it promotes immune activation and mediates antibody-dependent cellular cytotoxicity. Interestingly, the gene harbors a well-characterized protein altering polymorphism in the third exon (rs759550223, 57X/Q), which is in LD with the locus lead SNP (rs34881159, D′ = 0.7686, *P* = 0.026); the reference T allele introduces a premature stop codon yielding a nonfunctional receptor and thus contributes to interindividual variability in immune responses. This functional variation can influence the plasma N-glycome by modulating immune complex handling and antibody effector functions (Bournazos and Ravetch 2015; Frampton et al. 2024).

The *MGAT4B* encodes the enzyme N-acetylglucosaminyltransferase IVB (GnT-IVb), which catalyzes the formation of complex-type N-glycans. Its role in glycan biosynthesis is directly reflected in our results, as its top associations are with complex, highly branched N-glycans (CA3, H6N5E3, CA2) (Varki et al. 2022).

The *MAML1* gene is a key transcriptional co-activator in the Notch signaling pathway (Rampias et al. 2014). We suggest that *MAML1* may regulate the expression of specific glycosyltransferases. This represents a novel, indirect transcriptional mechanism for regulating glycosylation that warrants further investigation.

The gene *RNF168* (ring finger protein 168), replicated previously in multivariate IgG GWAS (Shadrina et al. 2021), encodes an E3 ubiquitin ligase protein involved in DNA double-strand break repair and immunoglobulin class switch recombination (Ramachandran et al. 2010). As far as we are aware, *RNF168* involvement in glycosylation processes has not been reported, but if so, a pleiotropic action on immune-related and glycosylation traits may be speculated.

The *C3* gene encodes the central component of the complement system. Its prioritization, alongside our previous identification of the complement regulator *CFH* (Sharapov et al. 2025), suggests a common mechanism where genetic variation affecting complement cascade activity indirectly shapes the plasma N-glycome, potentially through the regulation of inflammation (Herbert et al. 2015).

Prioritization of *C3* is of particular interest as this continues highlighting the role of the complement system, which has been implicated by the effects of *CFH* on the plasma N-glycome in a recent work of Sharapov et al. (Sharapov et al. 2025). *C3* is primarily expressed in hepatocytes, and the locus containing this gene is predominantly associated with oligomannose traits found in liver-derived proteins (Table 3). This association supports the regulation of N-glycosylation through liver biology.

To conclude, in the present study, we demonstrate that analysis of plasma N-glycome using MALDI-MS with linkage-specific sialic acid derivatization allows higher resolution and power in analysis of genetic associations with α2,3- and α2,6- sialylation (Table 2). This is especially important given the linkage-specific associations to metabolic and liver disease (Dotz et al. 2018; Blood N-glycomic signature of fibrosis in MASLD shows low levels of global α2,3-sialylation | medRxiv, 2026). Further, we extended the list of loci significantly and replicably associated with total plasma N-glycosylation by 10 (a more than 20% increase).

Deeper understanding of biological pathways involved in regulation of N-glycosylation through high-confidence genetic discoveries will facilitate the use of glycans and glycosylation pathways as therapeutic targets and biomarkers of human diseases.

## Materials and methods

### Study cohort description

This work is based on analyzing of the glycomic and genetic data from two cohorts — the DiaGene study (van Herpt et al. 2017) and the Hoorn Diabetes Care System (van der Heijden et al. 2017).

### DiaGene study

The DiaGene study is a multi-center case–control study of type 2 diabetes in the first and second line of care in the region of Eindhoven, the Netherlands. The aim of the DiaGene study was to unravel the etiology of type 2 diabetes and its complications. The DiaGene study includes 1886 patients with type 2 diabetes (mean age 65.3 years, SD 9.5; 50.7% female; mean BMI 28.0 kg/m^2^) and 854 controls without diabetes (mean age 66.7 years, SD 7.8; 53.5% female; mean BMI 26.1 kg/m^2^), of these, in 1490 cases and 544 controls, the total plasma N-glycome and genotypes were available for analysis. Virtually all patients with type 2 diabetes living in Eindhoven were approached for inclusion, between 2006 and 2011. The control group consisted of people without any kind of diabetes, Cushing’s disease or metformin use; aged 55 years or older (van Herpt et al. 2017).

### Hoorn diabetes care system

The Hoorn Diabetes Care System (Hoorn DCS) cohort consists of persons with T2D in primary care from the West-Friesland region of the Netherlands. Enrolment in the cohort started in 1998 and this prospective dynamic cohort currently holds 12,673 persons with T2D (mean age 61.1 years, SD 11.0; 44.6% female; median BMI 29.4 kg/m^2^), of these, in 1351 cases, the total plasma N-glycome and genotypes were available for analysis (van der Heijden et al. 2017).

### Mass spectrometry

Total plasma N-glycome analysis and mass spectrometry data processing for the DiaGene study was based on the work-flow from Reiding et al. (Reiding et al. 2014; Bladergroen et al. 2015) and is described by Dotz et al. (Dotz and Wuhrer 2019). In short, total plasma N-glycome was measured by MALDI-time-of-flight (TOF)-MS on a Bruker ultrafleXtreme instrument after enzymatic glycan release from plasma glycoproteins and linkage-specific sialic acid derivatization (Vreeker et al. 2018). Quality control of the mass-spectra and glycan analytes was performed, and samples were excluded in case of low intensity or interferences. Quality control of the mass-spectra and glycan analytes was performed using MassyTools, which calculates signal-to-noise ratios, mass accuracy (ppm), and isotopic pattern quality scores to enable automatic filtering of invalid peaks and background noise. Mass spectra were excluded from further analysis if their total intensity was lower than 50,000 and their “Fraction of analyte area - background area above signal-to-noise ratio” was lower than 0.89 (corresponding to the mean minus four-times the SD to ensure a sufficient intensity for the overall profile including minor peaks; see for further details on the quality parameters) (Jansen et al. 2015). In total, 2651 DiaGene study samples (1490 T2D cases and 544 controls) and 1351 samples (T2D cases) from the Hoorn Diabetes Care System cohort passed QC. Glycan peaks, representing quantitative measurements of glycan levels, were defined by manual or automatic integration of the corresponding intensity peaks in the chromatograms. In total, 73 direct traits passed quality control, and 91 derived traits were calculated by normalization to their sum for the DiaGene cohort; 68 direct and 86 derived traits were calculated for the Hoorn Diabetes Care System cohort. Batch correction was performed using the ComBat method (Johnson et al. 2007), adjusting for preparation day, MALDI plate, row, and column as covariates. The traits were harmonized between two datasets (see Supplementary Table 1a). Magnitude mode MALDI-FT-ICR-MS was performed on TPNG samples from five different healthy donors as described previously (Vreeker et al. 2018). Absorption mode mass spectra were generated using AutoVectis Pro version 21.0.1f2. Compositional assignment of the hexuronic acid-containing N-glycan was achieved by liquid chromatography-MS/MS after glycan labeling with 2-aminobenzamide (de Haan et al. 2022).

### Genotyping

#### DiaGene study

The DiaGene study participants were genotyped using the Illumina Global Screening array (GSA v1) and genotypes were imputed to the Haplotype Reference Consortium (HRC) reference panel (r1.1) (McCarthy et al. 2016) using the Michigan Imputation Server (Das et al. 2016).

#### Hoorn diabetes care system

In total 1800 Hoorn DCS samples are genotyped and have TPNG measured. All 1800 samples are of western European descent. 1600 T2D patients were genotyped using Illumina Core Exome SNP-array. 200 T2D-controls were genotyped using Affymetrix Axiom SNP-array. All genotypes were HRC imputed.

### Genetic association analysis

#### UHPLC-FD GWAS

We reused summary statistics from the replication cohort (TwinsUK, CEDAR, QMDiab and SABRE) (*n* = 3224) of univariate and multivariate genetic association analysis and meta-analysis of the association between 117 univariate and 21 multivariate total plasma N-glycome traits and the 19 sentinel variants tagging loci that have been reported at genome-wide significance, but did not replicate in the study of Sharapov et al. (Sharapov et al. 2025).

#### MALDI-MS GWAS

The genetic association analysis in each cohort was conducted using the same protocol as in Sharapov et al. (Sharapov et al. 2025). Briefly, prior to GWAS, the total plasma N-glycome traits were adjusted for sex and age, and the residuals were quantile transformed to normal distribution. We assumed an additive model of genetic effects. GWAS were based on the genotypes imputed from Haplotype Reference Consortium Results (McCarthy et al. 2016) or 1000 Genomes project (1000 Genomes Project Consortium et al. 2015). Results of univariate GWAS in DiaGene study and Hoorn DCS cohorts passed a strict quality control procedure that included standardized data aggregation, format unification, and quality filtering of GWAS summary statistics to ensure consistency and reliability for downstream analyses, followed by a fixed-effects inverse-variance weighted meta-analysis (*n* = 3385). In addition, we conducted a multivariate GWAS of the total plasma N-glycome on groups of N-glycome traits (Supplementary Table 2b) based on the biochemical similarity between glycans measured using MALDI-MS technology. The multivariate analysis was performed using the MANOVA-based method, adopted for analysis of a group of single-trait GWAS summary statistics (the MultiABEL R package) (Shen et al. 2017). We performed genetic association analyses on 143 univariate (Supplementary Table 2a) and 24 multivariate traits (Supplementary Table 2b).

#### Replication

Since we cannot fully match the N-glycan traits measured by UHPLC-FD and MALDI-MS, for each of the 19 glyQTLs not replicated in Sharapov et al. (Sharapov et al. 2025), we applied the Cauchy aggregation test (Liu and Xie 2020) to combine P-values across all glycan traits in UHPLC-FD and MALDI-MS N-glycome datasets:


\begin{align*} {T}_{UHPLC- FD}=\sum_{i=1}^{138}\frac{1}{138}\tan \left\{\left(0.5-{p}_i\right)\mathrm{\pi} \right\}\!, \end{align*}

\begin{align*} {T}_{MALDI- MS}=\sum_{i=1}^{167}\frac{1}{167}\tan \left\{\left(0.5-{p}_i\right)\pi \right\}\!, \end{align*}

where ${p}_i$ is the p-value for the association of the i-th glycan trait. P-values were combined using the CCT function of the R package STAAR, which aggregates p values using the Cauchy method. We conducted a one-sided Fisher’s combined probability test for meta-analysis (Brown 1975) of Cauchy aggregated p-values from UHPLC-FD and MALDI-MS datasets:


$$ {\mathrm{\chi}}_2^2=-2\times \left(\mathit{\ln}\left({p}_{UHPLC- FD}\right)+\mathit{\ln}\left({p}_{MALDI- MS}\right)\right)\!, $$

resulting in replication p-values (Supplementary table 3). A locus was considered replicated if the Fisher’s p-value exceeded the threshold of $0.05/19=2.63\times{10}^{-3}$, where 19 is the number of selected glyQTLs.

#### Annotation of glyQTLs

We annotated all 59 loci found previously in the study of Sharapov et al. (Sharapov et al. 2025) by calculating coefficient of determination R^2^ of association between a locus and two groups of traits – 117 UHPLC-FD-measured N-glycome traits (Supplementary Table 5a) as well as 143 MALDI-MS-measured N-glycome traits (Supplementary Table 5b). Glycan graphical representations following the recommendations of the Consortium for Functional Glycomics (CFG) are presented in Supplementary Table 2a, c, d.

### Prioritization of candidate genes in found loci

#### Functional annotation of genetic variants

We inferred the possible molecular consequences of genetic variants in glyQTLs. We focused on variants in LD with lead variants. We created a “long list” of putative causal variants using PLINK version 1.9 (−-show-tags option) (Purcell et al. 2007), applied to whole genome re-sequenced data for 503 European ancestry individuals (1000 Genomes phase 3 version 5 data). The size of the window to find the LD was equal to 500 kb. The default ${r}^2\ge 0.8$ value was taken as a threshold to include SNPs into the credible set. Prioritization of genes containing variants in strong LD with the lead variant, which are protein truncating variants (annotated by Variant Effect Predictor, VEP (McLaren et al. 2016)) (Supplementary Table 4b) or damaging according to FATHMM XF (Rogers et al. 2018) (Supplementary Table 4c), FATHMM InDel (Ferlaino et al. 2017) (Supplementary Table 4d).

#### Genes of N-glycan biosynthesis and congenital disorders of glycosylation

We searched for the genes encoding glycosyltransferases – enzymes with a known role in N-glycan biosynthesis (Narimatsu et al. 2019), located in the ±250 Kb-vicinity of the lead SNPs in glyQTLs. Additionally, we prioritized genes with known mutations that cause Congenital Disorders of Glycosylation according to MedGen database (https://www.ncbi.nlm.nih.gov/medgen/76469) located in the vicinity of ±250 kb from the lead SNPs.

#### Colocalization with eQTL and pQTL

To find potential pleiotropic effects of glyQTL on gene expression levels in relevant tissues, we applied Summary data-based Mendelian Randomization analysis followed by the Heterogeneity in Dependent Instruments (SMR/HEIDI) (Zhu et al. 2016) on expression of quantitative trait loci (eQTLs) obtained from Westra Blood eQTL collection (Westra et al. 2013) from peripheral blood; GTEx (version 7) eQTL collection (The G Consortium atlas of genetic regulatory effects across human tissues 2020) of liver and whole blood; CEDAR eQTL collection (Momozawa et al. 2018) from CD19+ B lymphocytes, CD8+ T lymphocytes, CD4+ T lymphocytes, CD14+ monocytes, and CD15+ granulocytes; and on protein quantitative trait loci (pQTLs) using SomaLogic datasets (Suhre et al. 2017; Sun et al. 2018). The outcome variable was the N-glycome trait with the most significant univariate association. If glyQTLs were only replicated in multivariate analyses, we used summary statistics for the most significantly associated univariate trait as the primary trait in the analysis.

The results of the SMR test were considered statistically significant if ${P}_{adj}<0.05$ (Benjamini-Hochberg adjusted *P*). The significance threshold for HEIDI tests was set at $P=0.05$ ($P<0.05$corresponds to the rejection of the pleiotropy hypothesis) (Supplementary Table 4f and g).

#### Depict

Gene prioritization and gene set and tissue/cell type enrichment analyses were performed using the Data-driven Expression Prioritized Integration for Complex Traits framework (DEPICT) (Pers et al. 2015) applied to GWAMA summary statistics for the samples of European descent (*n* = 10,172) (Sharapov et al. 2025). DEPICT analysis was conducted for SNPs associated with any N-glycosylation trait at $P<5\times{10}^{-8}/28$. The significance threshold for DEPICT analysis was set at False Discovery Rate $FDR<0.20$ (Supplementary Table 4e).

## Supplementary Material

Supplementary_Figures_cwag029

ST1_cwag029

ST2_cwag029

ST3_cwag029

ST4_cwag029

ST5_cwag029

Supplementary_Materials_cwag029

## Data Availability

UHPLC-FD: The first part of the full genome-wide summary association statistics for 117 glycome traits from the replication UHPLC-FD GWAMA (Genome-Wide Association Meta-Analysis) conducted on participants, totaling 3224 individuals, have been deposited in the Zenodo database under accession code 15161945 (CC BY 4.0) (https://doi.org/10.5281/zenodo.15161945). The second part of the full genome-wide summary association statistics for 117 glycome traits from the replication UHPLC-FD GWAMA (Genome-Wide Association Meta-Analysis) conducted on participants, totaling 3224 individuals, have been deposited in the Zenodo database under accession code 15166735 (CC BY 4.0) (https://doi.org/10.5281/zenodo.15166735). MALDI-MS: The full genome-wide summary association statistics the 57 direct and 86 derived glycome traits from the replication MALDI-MS GWAMA (N = 3,385) have been deposited in Zenodo (CC BY 4.0; (https://doi.org/10.5281/zenodo.19679098 and https://doi.org/10.5281/zenodo.19705289, respectively).

## References

[ref1] Abdellaoui A, Yengo L, Verweij KJH, Visscher PM. 15 years of GWAS discovery: realizing the promise. Am J Hum Genet. 2023:110(2):179–194. 10.1016/j.ajhg.2022.12.011. 36634672 PMC9943775

[ref2] Bladergroen MR, Reiding KR, Hipgrave Ederveen AL, Vreeker GCM, Clerc F, Holst S, Bondt A, Wuhrer M, van der Burgt YEM. Automation of high-throughput mass spectrometry-based plasma N-Glycome analysis with linkage-specific sialic acid esterification. J Proteome Res. 2015:14(9):4080–4086. 10.1021/acs.jproteome.5b00538. 26179816

[ref3] Blood N-glycomic signature of fibrosis in MASLD shows low levels of global α2,3-sialylation | medRxiv . 2026 [accessed 2024 Dec 24]. 10.1101/2024.09.19.24313949v1.

[ref4] Bournazos S, Ravetch JV. Fcγ receptor pathways during active and passive immunization. Immunol Rev. 2015:268(1):88–103. 10.1111/imr.12343. 26497515 PMC7556827

[ref5] Brown MB . 400: A method for combining non-independent, one-sided tests of significance. Biometrics. 1975:31(4):987–992. 10.2307/2529826.

[ref6] Clerc F, Reiding KR, Jansen BC, Kammeijer GSM, Bondt A, Wuhrer M. Human plasma protein N-glycosylation. Glycoconj J. 2016:33(3):309–343. 10.1007/s10719-015-9626-2. 26555091 PMC4891372

[ref7] Das S, Forer L, Schönherr S, Sidore C, Locke AE, Kwong A, Vrieze SI, Chew EY, Levy S, McGue M, et al. Next-generation genotype imputation service and methods. Nat Genet. 2016:48(10):1284–1287. 10.1038/ng.3656. 27571263 PMC5157836

[ref8] Dotz V, Wuhrer M. N-glycome signatures in human plasma: associations with physiology and major diseases. FEBS Lett. 2019:593(21):2966–2976. 10.1002/1873-3468.13598. 31509238

[ref9] Dotz V, Lemmers RFH, Reiding KR, Hipgrave Ederveen AL, Lieverse AG, Mulder MT, Sijbrands EJG, Wuhrer M, vanHoek M. Plasma protein *N-*glycan signatures of type 2 diabetes. Biochim Biophys Acta Gen Subj. 2018:1862(12):2613–2622. 10.1016/j.bbagen.2018.08.005. 30251656

[ref10] Ferlaino M, Rogers MF, Shihab HA, Mort M, Cooper DN, Gaunt TR, Campbell C. An integrative approach to predicting the functional effects of small indels in non-coding regions of the human genome. BMC Bioinformatics. 2017:18(1):442. 10.1186/s12859-017-1862-y. 28985712 PMC5955213

[ref11] Frampton S, Smith R, Ferson L, Gibson J, Hollox EJ, Cragg MS, Strefford JC. Fc gamma receptors: their evolution, genomic architecture, genetic variation, and impact on human disease. Immunol Rev. 2024:328(1):65–97. 10.1111/imr.13401. 39345014 PMC11659932

[ref12] Genomes Project Consortium, Auton A, Brooks LD, Durbin RM, Garrison EP, Kang HM, Korbel JO, Marchini JL, McCarthy S, McVean GA, et al. A global reference for human genetic variation. Nature. 2015:526(7571):68–74. 10.1038/nature15393. 26432245 PMC4750478

[ref13] de Haan N, Narimatsu Y, Koed Møller Aasted M, Larsen ISB, Marinova IN, Dabelsteen S, Vakhrushev SY, Wandall HH. In-depth profiling of O-glycan isomers in human cells using C18 Nanoliquid chromatography-mass spectrometry and Glycogenomics. Anal Chem. 2022:94(10):4343–4351. 10.1021/acs.analchem.1c05068. 35245040 PMC8928149

[ref14] van der Heijden AA, Rauh SP, Dekker JM, Beulens JW, Elders P, t Hart LM, Rutters F, vanLeeuwen N, Nijpels G. The Hoorn diabetes care system (DCS) cohort. A prospective cohort of persons with type 2 diabetes treated in primary care in the Netherlands. BMJ Open. 2017:7(5):e015599. 10.1136/bmjopen-2016-015599. PMC5729999 28588112

[ref15] Herbert AP, Makou E, Chen ZA, Kerr H, Richards A, Rappsilber J, Barlow PN. Complement evasion mediated by enhancement of captured factor H: implications for protection of self-surfaces from complement. J Immunol. 2015:195(10):4986–4998. 10.4049/jimmunol.1501388. 26459349 PMC4635569

[ref16] vanHerpt TTW, Lemmers RFH, vanHoek M, Langendonk JG, Erdtsieck RJ, Bravenboer B, Lucas A, Mulder MT, Haak HR, Lieverse AG, et al. Introduction of the DiaGene study: clinical characteristics, pathophysiology and determinants of vascular complications of type 2 diabetes. Diabetol Metab Syndr. 2017:9:47. 10.1186/s13098-017-0245-x. 28649285 PMC5477157

[ref17] Huffman JE, Knezevic A, Vitart V, Kattla J, Adamczyk B, Novokmet M, Igl W, Pucic M, Zgaga L, Johannson Å, et al. Polymorphisms in B3GAT1, SLC9A9 and MGAT5 are associated with variation within the human plasma N-glycome of 3533 European adults. Hum Mol Genet. 2011:20(24):5000–5011. 10.1093/hmg/ddr414. 21908519

[ref18] Jansen BC, Reiding KR, Bondt A, Hipgrave Ederveen AL, Palmblad M, Falck D, Wuhrer M. MassyTools: A high-throughput targeted data processing tool for relative quantitation and quality control developed for Glycomic and glycoproteomic MALDI-MS. J Proteome Res. 2015:14(12):5088–5098. 10.1021/acs.jproteome.5b00658. 26565759

[ref19] Johnson WE, Li C, Rabinovic A. Adjusting batch effects in microarray expression data using empirical bayes methods. Biostatistics. 2007:8(1):118–127. 10.1093/biostatistics/kxj037. 16632515

[ref20] Lachmann A, Torre D, Keenan AB, Jagodnik KM, Lee HJ, Wang L, Silverstein MC, Ma’ayan A. Massive mining of publicly available RNA-seq data from human and mouse. Nat Commun. 2018:9(1):1366. 10.1038/s41467-018-03751-6. 29636450 PMC5893633

[ref21] Lauc G, Trbojević-Akmačić I. The role of glycosylation in health and disease. Springer Nature; 2021.

[ref22] Liu Y, Xie J. Cauchy combination test: A powerful test with analytic p-value calculation under arbitrary dependency structures. J Am Stat Assoc. 2020:115(529):393–402. 10.1080/01621459.2018.1554485. 33012899 PMC7531765

[ref23] Maslov DE, Timoshchuk AN, Bondar AA, Golubev MP, Soplenkova AG, Hanic M, Sharapov SZ, Leonova ON, Aulchenko YS, Golubeva TS. Fast and simple protocol for N-Glycome analysis of human blood plasma proteome. Biomolecules. 2024:14(12):1551. 10.3390/biom14121551. 39766258 PMC11673551

[ref24] McCarthy S, Das S, Kretzschmar W, Delaneau O, Wood AR, Teumer A, Kang HM, Fuchsberger C, Danecek P, Sharp K, et al. A reference panel of 64,976 haplotypes for genotype imputation. Nat Genet. 2016:48(10):1279–1283. 10.1038/ng.3643. 27548312 PMC5388176

[ref25] McLaren W, Gil L, Hunt SE, Riat HS, Ritchie GRS, Thormann A, Flicek P, Cunningham F. The Ensembl variant effect predictor. Genome Biol. 2016:17(1):122. 10.1186/s13059-016-0974-4. 27268795 PMC4893825

[ref26] Momozawa Y, Dmitrieva J, Théâtre E, Deffontaine V, Rahmouni S, Charloteaux B, Crins F, Docampo E, Elansary M, Gori A-S, et al. IBD risk loci are enriched in multigenic regulatory modules encompassing putative causative genes. Nat Commun. 2018:9(1):2427. 10.1038/s41467-018-04365-8. 29930244 PMC6013502

[ref27] Narimatsu Y, Joshi HJ, Nason R, Van Coillie J, Karlsson R, Sun L, Ye Z, Chen Y-H, Schjoldager KT, Steentoft C, et al. An atlas of human glycosylation pathways enables display of the human Glycome by gene engineered cells. Mol Cell. 2019:75(2):394–407.e5. 10.1016/j.molcel.2019.05.017. 31227230 PMC6660356

[ref28] Pers TH, Karjalainen JM, Chan Y, Westra H-J, Wood AR, Yang J, Lui JC, Vedantam S, Gustafsson S, Esko T, et al. Biological interpretation of genome-wide association studies using predicted gene functions. Nat Commun. 2015:6:5890. 10.1038/ncomms6890. 25597830 PMC4420238

[ref29] Purcell S, Neale B, Todd-Brown K, Thomas L, Ferreira MAR, Bender D, Maller J, Sklar P, de Bakker PIW, Daly MJ, et al. PLINK: a tool set for whole-genome association and population-based linkage analyses. Am J Hum Genet. 2007:81(3):559–575. 10.1086/519795. 17701901 PMC1950838

[ref30] Ramachandran S, Chahwan R, Nepal RM, Frieder D, Panier S, Roa S, Zaheen A, Durocher D, Scharff MD, Martin A. The RNF8/RNF168 ubiquitin ligase cascade facilitates class switch recombination. Proc Natl Acad Sci U S A. 2010:107(2):809–814. 10.1073/pnas.0913790107. 20080757 PMC2818930

[ref31] Rampias T, Vgenopoulou P, Avgeris M, Polyzos A, Stravodimos K, Valavanis C, Scorilas A, Klinakis A. A new tumor suppressor role for the notch pathway in bladder cancer. Nat Med. 2014:20(10):1199–1205. 10.1038/nm.3678. 25194568

[ref32] Reiding KR, Blank D, Kuijper DM, Deelder AM, Wuhrer M. High-throughput profiling of protein N-glycosylation by MALDI-TOF-MS employing linkage-specific sialic acid esterification. Anal Chem. 2014:86(12):5784–5793. 10.1021/ac500335t. 24831253

[ref33] Reiding KR, Bondt A, Hennig R, Gardner RA, O’Flaherty R, Trbojević-Akmačić I, Shubhakar A, Hazes JMW, Reichl U, Fernandes DL, et al. High-throughput serum N-Glycomics: method comparison and application to study rheumatoid arthritis and pregnancy-associated changes *[S]. Mol Cell Proteomics. 2019:18(1):3–15. 10.1074/mcp.RA117.000454. 30242110 PMC6317482

[ref34] Reily C, Stewart TJ, Renfrow MB, Novak J. Glycosylation in health and disease. Nat Rev Nephrol. 2019:15(6):346–366. 10.1038/s41581-019-0129-4. 30858582 PMC6590709

[ref35] Rogers M, Shihab HA, Mort M, Cooper D, Gaunt TR, Campbell C. FATHMM-XF: accurate prediction of pathogenic point mutations via extended features. Bioinform. 2018:34(3):511–513. 10.1093/bioinformatics/btx536. PMC5860356 28968714

[ref36] Shadrina AS, Zlobin AS, Zaytseva OO, Klarić L, Sharapov SZ, Pakhomov DE, Perola M, Esko T, Hayward C, Wilson JF, et al. Multivariate genome-wide analysis of immunoglobulin G N-glycosylation identifies new loci pleiotropic with immune function. Hum Mol Genet. 2021:30(13):1259–1270. 10.1093/hmg/ddab072. 33710309

[ref37] Sharapov SZ, Shadrina AS, Tsepilov YA, Elgaeva EE, Tiys ES, Feoktistova SG, Zaytseva OO, Vuckovic F, Cuadrat R, Jäger S, et al. Replication of 15 loci involved in human plasma protein N-glycosylation in 4802 samples from four cohorts. Glycobiology. 2021:31(2):82–88. 10.1093/glycob/cwaa053. 32521004 PMC7874387

[ref38] Sharapov S, Timoshchuk A, Zaytseva O, Maslov DE, Soplenkova A, Elgaeva EE, Tiys ES, Mangino M, Wittenbecher C, Karssen L, et al. A genome-wide association study in 10,000 individuals links plasma N-glycome to liver disease and anti-inflammatory proteins. Nat Commun. 2025:16(1):5525. 10.1038/s41467-025-60431-y. 40593539 PMC12218978

[ref39] Shen X, Klarić L, Sharapov S, Mangino M, Ning Z, Wu D, Trbojević-Akmačić I, Pučić-Baković M, Rudan I, Polašek O, et al. Multivariate discovery and replication of five novel loci associated with immunoglobulin G N-glycosylation. Nat Commun. 2017:8(1):447. 10.1038/s41467-017-00453-3. 28878392 PMC5587582

[ref40] Smith KGC, Clatworthy MR. FcgammaRIIB in autoimmunity and infection: evolutionary and therapeutic implications. Nat Rev Immunol. 2010:10(5):328–343. 10.1038/nri2762. 20414206 PMC4148599

[ref41] Suhre K, Arnold M, Bhagwat AM, Cotton RJ, Engelke R, Raffler J, Sarwath H, Thareja G, Wahl A, DeLisle RK, et al. Connecting genetic risk to disease end points through the human blood plasma proteome. Nat Commun. 2017:8:14357. 10.1038/ncomms14357. 28240269 PMC5333359

[ref42] Sun BB, Maranville JC, Peters JE, Stacey D, Staley JR, Blackshaw J, Burgess S, Jiang T, Paige E, Surendran P, et al. Genomic atlas of the human plasma proteome. Nature. 2018:558(7708):73–79. 10.1038/s41586-018-0175-2. 29875488 PMC6697541

[ref43] The GTEx consortium . Atlas of genetic regulatory effects across human tissues. Science. 2020:369(6509):1318–1330. 10.1126/science.aaz1776. 32913098 PMC7737656

[ref44] Timoshchuk A, Sharapov S, Aulchenko YS. Twelve years of genome-wide association studies of human protein N-glycosylation. Engineering. 2023:26:17–31. 10.1016/j.eng.2023.03.013.

[ref45] Varki A, Cummings RD, Esko JD, Stanley P, Hart GW, Aebi M, Mohnen D, Kinoshita T, Packer NH, Prestegard JH, et al. Essentials of glycobiology. 4th ed. Cold Spring Harbor (NY): Cold Spring Harbor Laboratory Press; 2022. [accessed 2024 Dec 24]. http://www.ncbi.nlm.nih.gov/books/NBK579918/35536922

[ref46] Vreeker GCM, Nicolardi S, Bladergroen MR, van der Plas CJ, Mesker WE, Tollenaar RAEM, van der Burgt YEM, Wuhrer M. Automated plasma Glycomics with linkage-specific sialic acid esterification and ultrahigh resolution MS. Anal Chem. 2018:90(20):11955–11961. 10.1021/acs.analchem.8b02391. 30230816 PMC6209171

[ref47] Westra H-J, Peters MJ, Esko T, Yaghootkar H, Schurmann C, Kettunen J, Christiansen MW, Fairfax BP, Schramm K, Powell JE, et al. Systematic identification of trans eQTLs as putative drivers of known disease associations. Nat Genet. 2013:45(10):1238–1243. 10.1038/ng.2756. 24013639 PMC3991562

[ref48] Zhu Z, Zhang F, Hu H, Bakshi A, Robinson MR, Powell JE, Montgomery GW, Goddard ME, Wray NR, Visscher PM, et al. Integration of summary data from GWAS and eQTL studies predicts complex trait gene targets. Nat Genet. 2016:48(5):481–487. 10.1038/ng.3538. 27019110

