# Supplementary material for: Replication of 10 novel loci involved in human plasma protein N-glycosylation using MALDI-MS and UHPLC-FD data
Source: Glycobiology. 2026 Apr 20;36(6):cwag029. doi: 10.1093/glycob/cwag029 (PMC13159552; doi:10.1093/glycob/cwag029)
Supplement: Supplementary_Figures_cwag029 [file supplementary_figures_cwag029.pdf]

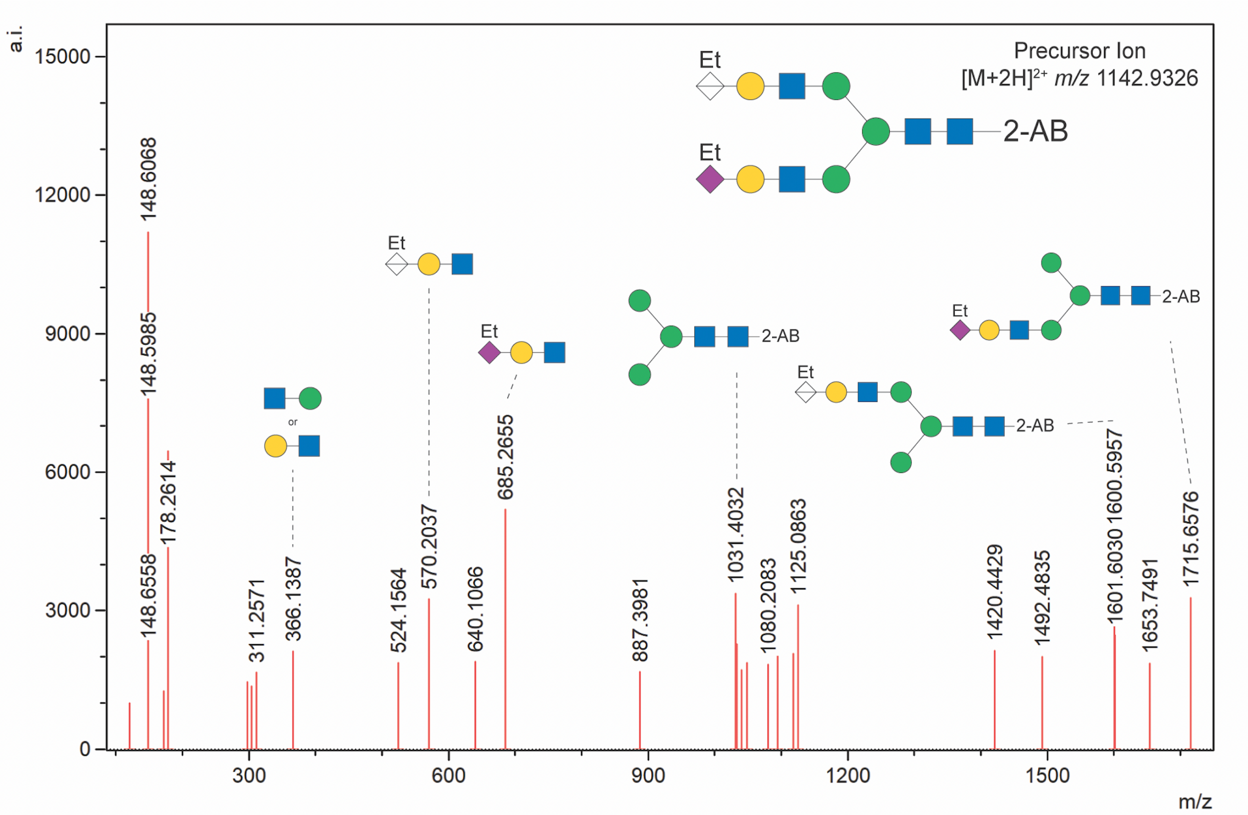

Supplementary Figure S1. Tandem MS spectrum of the 2AB-labeled, hexuronic acid-containing glycan H5N4E1G1. The 2AB-labeled glycan at m/z 1142.93 [M+2H]2+ with two ethyl-esterified carboxylic acid groups was subjected to collision-induced decay. Blue square, N-acetylglucosamine; green circle, mannose; yellow circle, galactose; purple diamond, sialic acid; white diamond, hexuronic acid (presumably glucuronic acid). Et indicates ethyl esterification.

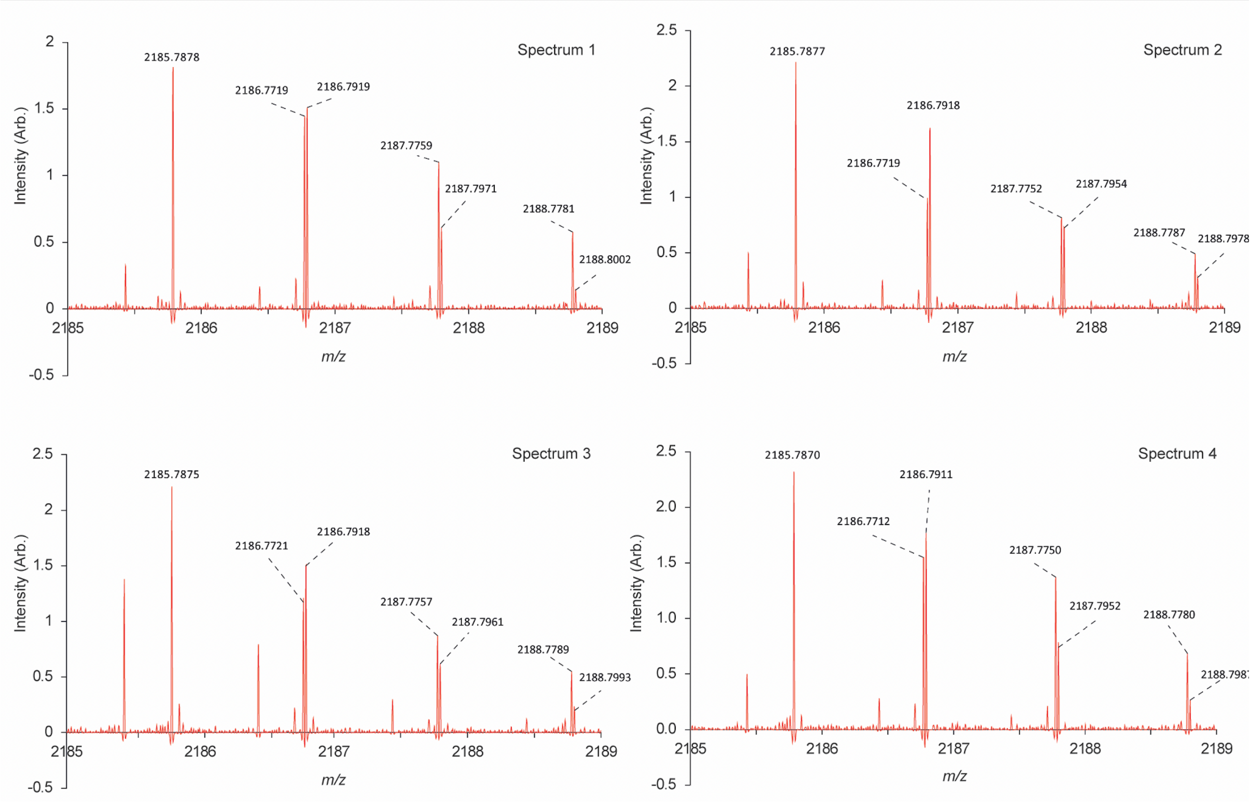

Supplementary Figure S2. MS evidence for a hexuronic acid-containing N-glycan in TPNG. Zoom-in regions of TPNG spectra are shown with two fully resolved glycan species in the region between m/z 2185 and m/z 2189. In all four spectra, anethyl-esterified 2,6-sialylated H5N5E1 N-glycan was detected at m/z 2185.7874 while an ethyl-esterified hexuronic acid-containing species (i.e. H5N4E1G1) was detected at m/z2186.7710. Blue square, N-acetylglucosamine; green circle, mannose; yellow circle, galactose; purple diamond, sialic acid; white diamond, hexuronic acid (presumably glucuronic acid). Et indicates ethyl esterification.
